# Operational Implementation of LED Fluorescence Microscopy in Screening Tuberculosis Suspects in an Urban HIV Clinic in Uganda

**DOI:** 10.1371/journal.pone.0072556

**Published:** 2013-09-06

**Authors:** Heidi Albert, Lydia Nakiyingi, Joseph Sempa, Olive Mbabazi, Sheena Mukkada, Barnabas Nyesiga, Mark D. Perkins, Yukari C. Manabe

**Affiliations:** 1 Foundation for Innovative New Diagnostics (FIND), Kampala, Uganda; 2 Infectious Diseases Institute, Makerere University College of Health Sciences, Kampala, Uganda; 3 Foundation for Innovative New Diagnostics (FIND), Geneva, Switzerland; 4 Division of Infectious Diseases, Department of Medicine, Johns Hopkins University School of Medicine, Baltimore, Maryland, United States of America; San Francisco General Hospital, University of California San Francisco, United States of America

## Abstract

**Background:**

Light emitting diode (LED) fluorescence microscopy (FM) is an affordable, technology targeted for use in resource-limited settings and recommended for widespread roll-out by the World Health Organization (WHO). We sought to compare the operational performance of three LED FM methods compared to light microscopy in a cohort of HIV-positive tuberculosis (TB) suspects at an urban clinic in a high TB burden country.

**Methods:**

Two spot specimens collected from TB suspects were included in the study. Smears were stained using auramine O method and read after blinding by three LED-based FM methods by trained laboratory technicians in the Infectious Diseases Institutelaboratory. Leftover portions of the refrigerated sputum specimens were transported to the FIND Tuberculosis Research Laboratory for Ziehl Neelsen (ZN) smear preparation and reading by experienced technologist as well as liquid and solid culture.

**Results:**

174 of 627 (27.8%) specimens collected yielded one or more positive mycobacterial cultures. 94.3% (164/174) were *M. tuberculosis* complex. LED FM was between 7.3–11.0% more sensitive compared to ZN microscopy. Of the 592 specimens examined by all microscopy methods, there was no significant difference in sensitivity between the three LED FM methods. The specificity of the LED FM methods was between 6.1% and 7.7% lower than ZN microscopy (*P*<0.001), although exclusion of the single poor reader resulted in over 98% specificity for all FM methods.

**Conclusions:**

Laboratory technicians in routine settings can be trained to use FM which is more sensitive than ZN microscopy. Despite rigorous proficiency testing, there were operator-dependent accuracy issues which highlight the critical need for intensive quality assurance procedures during LED FM implementation. The low sensitivity of FM for HIV-positive individuals particularly those with low CD4 T cell counts, will limit the number of additional patients found by LED FM in countries with high rates of HIV co-infection.

## Introduction

Fluorescence microscopy (FM) using auramine O staining for the detection of mycobacteria has been used for decades [1], but has been limited by the mercury vapour lamp (MVL) technology used in conventional FM which has an expensive power suppply, is inefficient, short-lived and has the potential to release toxic mercury [2]. Overall, in a meta-analysis published by Steingart and colleagues, FM is approximately 10% more sensitive than conventional light microscopy (LM) using Ziehl-Neelsen staining [3]. FM is also more efficient because slides can be read at lower magnification and requires a shorter examination time per slide [4]. The staining protocol is also easier and more efficient as it requires fewer reagents and steps than ZN staining.The advent of light emitting diode (LED) technology that is affordable for resource-limited settings, has a long lifespan of up to 50,000 hours, is efficient and has increased sensitivity [5], [6] led to the World Health Organization (WHO) recommendation that LED microscopy be phased in as an alternative to ZN [7]. In a comparison of the available microscopy options (LED FM, MVL FM and LM) against culture (gold standard) in 221 specimens (36 culture-positive for *Mycobacterial tuberculosis*), the sensitivity of the 3 techniques was not significantly different [8].

We previously evaluated the available commercialized LED microscope add-ons (Fraen AFTER™, Lumin™) and a stand-alone combination light and fluorescent microscope (Primostar™ iLED, Carl Zeiss) in a specialized TB laboratory using experienced technicians. We found that LED FM methods were more sensitive than LM and that examination time was shorter by all three LED methods. In addition, clear differences and operator preferences were noted with regard to installation, light intensity, contrast, need for darkroom, and availability of battery power supply for use in rural settings without consistent access to power [9]. The iLED was preferred for overall handling and features especially with regard to the homogeneity of illumination. Other side-by-side comparisons found Fraen superior to Lumin [10], with no difference observed between Lumin and iLED in a high income, low incidence experienced laboratory setting [11]. We sought to compare the operational performance of the three LED FM methods in a cohort of HIV-positive TB suspects at a busy urban clinic in Kampala, Uganda, a high burden TB country.

## Methods

### Ethical considerations

This protocol was reviewed and approved by the Scientific Review Committee of the Infectious Diseases Institute (IDI), the Institutional Review Boards of Makerere University, and the Uganda National Council for Science and Technology as previously described [9].The requirement for obtaining consent from patients was specifically waived by the institutional review boards because only anonymous sputum specimens, collected as part of routine clinical care were used and no identifying characteristics of patients were recorded or available to the laboratory technicians. The results of all testing were made available to clinicians for clinical care use, however. This data became part of the routinely collected database from which de-identified data for this study was also extracted and analyzed (CD4 T cell counts) and which is approved through the same ethical bodies listed above and previously described [12].

### Study setting and patient population

The first phase of the study was performed on sputum discards from Mulago Hospital and previously published [9]. This phase of the study was performed at the Adult Infectious Diseases Clinic (AIDC) at the IDI, Makerere University College of Health Sciences in Kampala. The AIDC has provided outpatient care to over 28,000 registered HIV-positive patients since its inception in 2002. Currently more than 10,000 patients are in active follow-up, over 7,000 of whom have been initiated on ART [13]. The IDI is a research and clinical center of excellence located adjacent to the Mulago Hospital, a tertiary referral government hospital. The IDI clinic laboratory where the FM was performed is located adjacent to the Makerere University- Johns Hopkins University clinical core laboratory which is College of American Pathologist certified.

Available investigations for TB include fluorescence microscopy, chest radiology, abdominal ultrasonography, and fine-needle aspiration of lymphadenopathy for acid fast bacilli microscopy. Diagnosis of TB is sometimes made on the basis of these investigations, but frequently on clinical suspicion alone. No mycobacterial culture facilities are available for routine evaluation. Patients diagnosed with TB are treated according to the Uganda National Tuberculosis Treatment Guidelines. Patients requiring inpatient care are referred to Mulago National Referral Hospital, a tertiary care hospital in the same complex.

Patients seeking outpatient care at the AIDC who present with TB signs and symptoms, are referred to the IDI TB Clinic, a separate, outdoor clinic for integrated TB and HIV care, for investigation and follow-up [12]. All care and treatment at the IDI is free of charge.

### Specimen collection and processing

Sputum specimens were collected from consecutive TB suspects presenting to the IDI TB clinic from June 2009- January 2010 in a cross-sectional study design. Patients were included per WHO intensive case finding guidelines if they had cough for 2 weeks or longer, fever, night sweats, or unintentional weight loss and were able to produce a sputum specimen. Patients presenting for treatment follow-up were excluded.

Three specimens are routinely collected for TB investigations: two spot and one early morning specimen (spot-morning-spot). The two spot specimens were included in the study. For purposes of this study, the early morning specimen was excluded due to concerns over specimen contamination and long transit time to the laboratory since culture was being performed as gold standard for this study. Specimens produced in the clinic were kept in a cool box until transfer to a refrigerator upon receipt at the laboratory. Two smears were prepared at IDI laboratory, stained using the auramine-O method, and stored in non-translucent slide boxes. One stained slide was transported to Mulago Microbiology laboratory for examination and reporting of the result. The second slide was retained at IDI for blinding and examination by three LED-based FM methods to be read at the IDI on the same day.

Following smear preparation, leftover portions of the sputum specimens were transported to the FIND Tuberculosis Research Laboratory which is situated at the National Tuberculosis Reference Laboratory. Mycobacteria Growth Indicator Tube (MGIT) and Lowenstein-Jensen (LJ) culture were performed according to standard methods with Capilia TB-Neo assay (Tauns Laboratories Inc. Namazu, Japan) used for *M.tuberculosis* complex identification. One additional direct smear per specimen was prepared for ZN stain and read at the research laboratory.

Staining reagents were prepared at the research laboratory and filtered on a weekly basis prior to use. Positive and negative control slides were included in each batch of test slides. Reading of the FM smear from one specimen using each LED FM method was performed by a single technologist. Therefore an over-labelling (blinding) system was implemented by a study coordinator, who was not involved in slide reading, to avoid interpretation bias.

### Microscopy methods

The following LED-based systems were evaluated: iLED Primostar (Zeiss), Fraen AFTER add-on device (Fraen Corporation), attached to Olympus X31 microscope, and Lumin add-on device (LW Scientific), attached to Olympus X31 microscope. Ziehl-Neelsen stained slides were read using the iLED light microscope. The conventional fluorescence slides were read using the NIKON Eclipse E200 mercury vapour lamp powered microscope at Mulago Hospital.

The order of reading of the three LED methods was alternated with each batch of slides (*e.g.*day 1: iLED, Fraen, Lumin; day 2: Fraen, Lumin, iLED; day 3: Lumin, Fraen, iLED), in order to minimize bias due to fluorescence extinction. Slides were not re-stained for reading in between the different LED-based techniques. Fluorescent smears were read at x40 magnification. Grading of smears was done according to WHO/IUATLD guidelines.

### Culture and identification

As previously described [9], after smear preparation, sputum was decontaminated by standard NALC-NaOH procedure (1.5% NaOH final concentration). Following neutralization and centrifugation the pellet was suspended in 1ml phosphate buffer pH6.8. 0.5 ml was used to inoculate MGIT culture and 0.1 ml to inoculate LJ culture. Positive cultures were identified using Capilia TB-Neo test. Capilia TB-Neo negative isolates were speciated using Genotype CM assay (Hain Lifescience, Nehren, Germany).

### Quality assurance

Each reader in the IDI stat laboratory was trained in a standardized 5-day fluorescence microscopy training to use all 3 of the microscopes, and to prepare auramine stain. After training, each reader examined a blinded panel of 30 slides each by ZN and by FM prior to the start of reading smears from clinical specimens. Acceptable performance comprised no high false (HF) results, no low false (LF) results and less than or equal to 3 quantification errors (QE). Acceptable performance in the panel slide set (competency testing) was a pre-requisite to starting the study according to the methods used in the previous study [9].

Routine FM at Mulago Hospital Microbiology laboratory was not subject to any training intervention or quality assurance procedures by the study team.

### Data analysis

Sensitivity and specificity (95% CI) were calculated for each method using culture as gold standard. The sensitivity and specificity of the methods were compared in a pairwise fashion and McNemar's test for equality of proportions for paired samples was used to determine whether the proportions of positive and negative results were the same for each method, using a 5% significance level. All data analysis was performed using STATA 11.0 software (Statcorp, College Station, TX, USA).

## Results

### Study population

**Figure 1:**
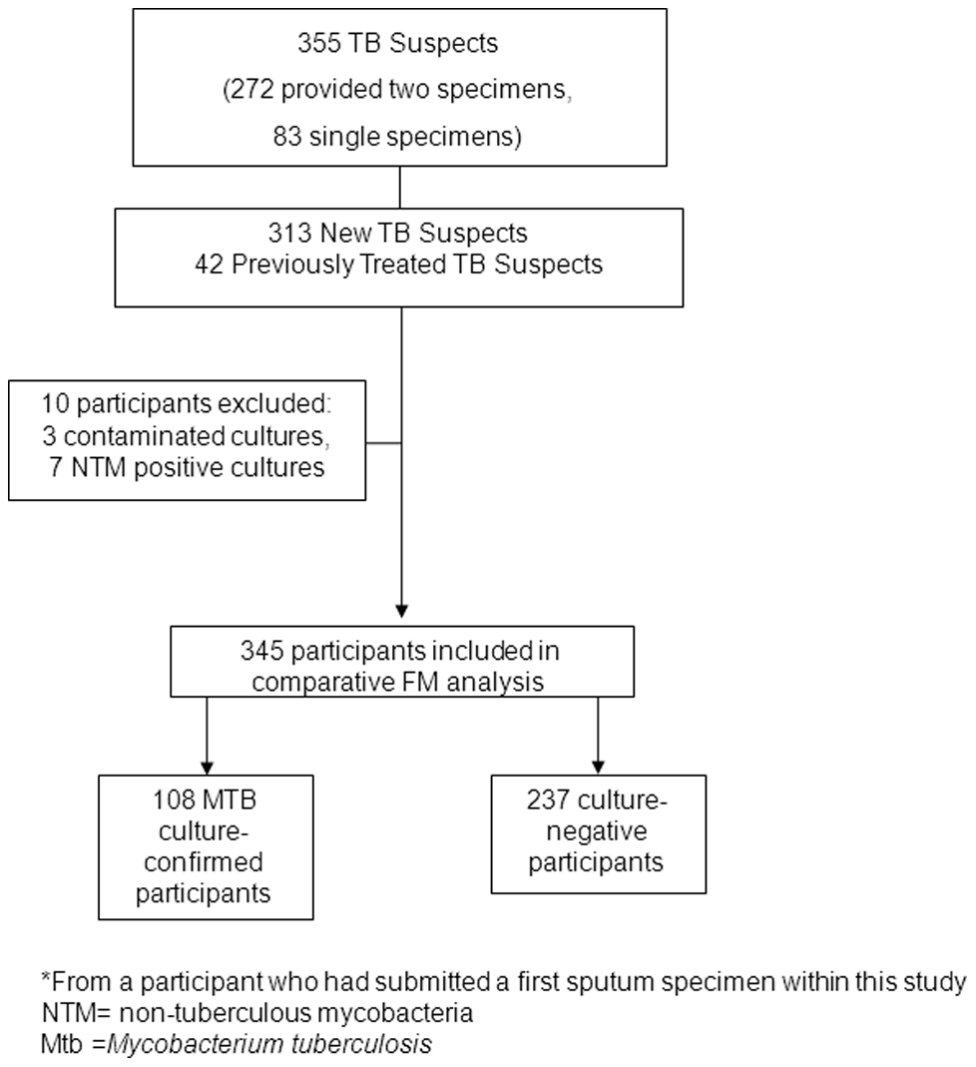
Diagram of study flow. NTM =  non-tuberculous mycobacteria, Mtb  =  *Mycobacterium tuberculosis*.

Three hundred and fifty-five HIV-infected study participants were enrolled in the study (Figure 1): 56.3% were females, median age 37.9 years (range of 31–44), the median CD4 was 294 cells/uL (IQR 148–481), and 63.9% were taking ART. Three hundred thirteen participants were new TB suspects, 42 had been previously treated for TB. The ZN microscopy positivity rate was [11% (39/355)] overall; 10.5 % (33/313) new TB suspects and 14.3% (6/42) in previously treated TB suspects. Of the 355 participants, a total of 115/355 (32.4%) had one or more positive mycobacterial cultures; 95/355 (26.8 %) were positive by MGIT culture (of which 65.3% (62/95) were also positive on solid culture). An additional 20 patients were positive on solid culture, but negative (11/20) or contaminated (9/20) on MGIT culture. Of the mycobacterial culture positive participants, 93.9% (108/115) were *M.tuberculosis* complex and 6.1% (7/115) were non-tuberculous mycobacteria (NTMs) (3 *M.intracellulare*, 1 *M. avium*, and 3 other mycobacteria).

### Specimens Analyzed

Six hundred and twenty-seven sputum specimens were included in the study. 550 specimens were from new TB suspects and 77 specimens were from previously treated suspects.The ZN microscopy positivity rate was 8.9% (56/627) overall; 8.2% (45/550) in specimens from new TB suspects and 14.3% (11/77) in specimens from previously treated TB suspects. Of the 627 sputum specimens, a total of 174/627(27.8%) specimens yielded one or more positive mycobacterial cultures; 151/627 (24.1%) were positive by MGIT culture (of which 103 were also positive on solid culture). An additional 23 specimens were positive on solid culture, but negative (12) or contaminated (11) on MGIT culture. Of the mycobacterial positive cultures, 94.3% (164/174) were *M.tuberculosis* complex (26.2% of all specimens) and 5.7% (10/174) were non-tuberculous mycobacteria (NTMs) (3 *M. intracellulare*, 1 *M.avium*, 2 *M.fortuitum* and 4 other mycobacteria).

### Performance of LED FM: per specimen and per patient analyses

Of the 627 specimens, 35 were excluded from the FM analysis (23 had contaminated culture results, 10 had cultures containing NTM and 2 specimens did not have slides available for all microscopy methods), leaving a total of 592 specimens. Of these, 164 had cultures positive for *M.tuberculosis* complex and were used for analysis.Results of routine ZN, routine FM, and LED FM are shown in Table 1.The sensitivity of LED FM by any of the three methods was between 7.3 and 11.0 % higher than ZN microscopy and between 5.5% and 9.2% higher than routine FM microscopy (mercury vapour lamp fluorescence microscopy at Mulago National Referral Hospital). The difference in sensitivity between LED FM and ZN was significant by all three methods (Fraen *P* = 0.002, iLED *P* = 0.011, Lumin *P* = 0.04). There was no significant difference in sensitivity between the three LED FM methods when compared in a pair-wise fashion.The specificity of the LED FM methods was between 6.1% and 7.7% lower than ZN microscopy and between 5.9% and 7.5% lower than routine FM microscopy and was significantly different for all methods compared with FM or ZN? (*P*<0.001).

**Table 1:** Performance of three LED-based fluorescence microscopy devices in detection of tuberculosis in HIV-positive suspects in Kampala, Uganda, per specimen analysis.

	ZN	Routine FM	iLED	Fraen	Lumin
**Sensitivity** *	31.1% (51/164) 24.1–38.8	32.9% (54/164) 25.8–40.7	40.2% (66/164) 32.7–48.2	42.1% (69/164) 34.4–50.0	38.4% (63/164) 30.9–46.3
3+	9		9	11	12
2+	13		20	18	20
1+	17		25	24	19
Scanty	12		11	16	12
No grading	0		1	0	0
**Specificity** *	99.3% (425/428) 98.0–99.9	99.1% (424/428) 97.6–99.7	93.2% (399/428) 90.4–95.4	91.6% (392/428) 88.5–94.0	91.6% (392/428) 88.5–94.0
**PPV**	94.4% (51/54) 84.6–98.8	93.1% (54/58) 83.3–98.1	69.5% (66/95) 59.2–78.5	65.7% (69/105) 55.8–74.7	63.6% (63/99) 53.4–73.1
**NPV**	79.0% (425/538) 75.3–82.4	79.4% (424/534) 75.7–82.8	80.3% (399/428) 76.3–84.0	80.5% (392/487) 76.7–83.9	79.5% (392/493) 75.7–83.0

* For sensitivity calculations, the denominator is the number of specimens for which the culture was positive for MTB. For specificity, the denominator shows the number of culture negative specimens.

Of the 355, the cultures of 10 participants were contaminated (n = 3) or positive for NTM (n = 7) and were excluded, leaving 345 participants eligible for the per patient accuracy analysis, of whom 108 had cultures positive for *M.tuberculosis* complex (Figure 1). Results of routine ZN, routine FM, and LED FM are shown in Table 2. As in the per specimen analysis, there was equivalent sensitivity between ZN and routine FM in the per patient analysis. The sensitivity of LED FM was significantly higher and specificity significantly lower than ZN microscopy and routine FM microscopy. There was no significant difference in sensitivity or specificity between the three LED FM methods when compared in a pair-wise fashion.

**Table 2:** Performance of three LED-based fluorescence microscopy devices in detection of tuberculosis in HIV-positive suspects in Kampala, Uganda, per patient analysis.

	ZN	Routine FM	iLED	Fraen	Lumin
**Sensitivity** *	32.4% (35/108) 23.7–42.0	32.4% (35/108) 23.7–42.0	44.4% (48/108) 34.9–54.3	43.5% (47/108) 34.0–53.3	40.7% (44/108) 31.3–50.6
3+	5		4	5	7
2+	7		15	13	16
1+	13		17	18	11
Scanty	10		11	11	10
No grading	0		1	0	0
**Specificity** *	98.7% (234/237) 96.3–99.7	99.1% (235/237) 97.0–99.9	89.5% (212/237) 84.8–93.0	87.0% (206/237) 82.0–90.9	88.1% (209/237) 83.3–92.0
**PPV**	92.1% (35/38) 78.6–98.3	94.6% (35/37) 81.8–99.3	65.8% (48/73) 53.7–76.5	60.2% (47/78) 48.5–71.1	61.1% (44/72) 48.9–72.3
**NPV**	76.2% (234/307) 71.0–80.9	76.3% (235/308) 71.2–80.9	78.0% (212/272) 72.5–82.7	77.1% (206/267) 71.6–82.0	76.6% (219/273) 71.0–81.5

* For sensitivity calculations, the denominator is the number of patients for which the culture was positive for MTB. For specificity, the denominator shows the number of culture negative patients. None of the 3 LED FM methods was significantly different from each other. ZN, ZiehlNeelsen staining method; FM, fluorescence microscopy; PPV, positive predictive value; NPV, negative predictive value.

### Per reader analysis of performance of LED FM

In a more in-depth analysis, the three readers for routine ZN (readers 1, 2, and 3) at the research laboratory showed significant inter-reader variability for ZN and, similarly inter-reader variability was observed in the 3 readers of the LED FM (readers 4, 5, and 6) by all 3 methods (Table 3). It was interesting to note that the sensitivity and specificity between LED FM methods did not vary significantly, and that the relative sensitivity of readers was maintained across methods; the same reader had the lowest sensitivity and highest specificity by all 3 LED methods. Similarly, the same reader had the highest sensitivity and lowest specificity by all 3 LED methods.

**Table 3:** Per reader analysis of performance of ZN and LED-based fluorescence microscopy methods, per specimen analysis.

	Overall	Reader 1	Reader 2	Reader 3	Reader 4	Reader 5	Reader 6
Direct ZN Sensitivity	31.1% (51/164) 24.1–38.8	40% (4/10) 12.2–73.8	35.9% (23/64) 24.3–48.9	26.7% (24/90) 17.9–37.0	-	-	-
Direct ZN Specificity	99.3% (425/428) 98.0–99.9	95.4% (41/43) 84.2–99.4	100.0% (125/125) 97.1–100	99.6% (259/260) 97.9–100	-	-	-
Direct ZN PPV	94.4% (51/54) 84.6–98.8	66.7% (4/6) 22.3–95.7	100.0% (23/23) 85.2–100	96.0% (24/25) 79.6–99.9	-	-	-
Direct ZN NPV	79.0% (425/538) 75.3–82.4	87.2% (41/47) 74.3–95.2	75.3% (125/166) 68.0–81.7	78.6% (259/325) 74.9–83.9	-	-	-
iLED Sensitivity	40.2% (66/164) 32.7–48.2	-	-	-	45.3% (34/75) 33.8–57.2	40.9% (9/22) 20.7–63.6	34.3% (23/67) 23.2–46.9
iLED Specificity	93.2% (399/428) 90.4–95.4	-	-	-	85.3% (156/183) 79.2–90.0	98.2% (54/55) 90.3–100	99.5% (189/190) 97.1–100
iLED PPV	69.5% (66/95) 59.2–78.5	-	-	-	55.7% (34/61) 42.4–68.5	90.0% (9/10) 55.5–99.7	95.8% (23/24) 78.9–99.9
iLED NPV	80.3% (399/497) 76.3–84.03	-	-	-	79.2% (156/197) 72.8–84.6	80.6% (54/67) 69.1–89.2	81.1% (189/233) 75.4–85.9
Fraen Sensitivity	42.1% (69/164) 34.4 50.0	-	-	-	49.3% (37/75) 37.6–61.1	40.9% (9/22) 20.7–63.6	34.3% (23/67) 23.2–46.9
Fraen Specificity	91.6% (392/428) 88.5–94.0	-	-	-	82.5% (151/183) 76.2–87.7	98.2% (54/55) 90.2–100	98.4% (187/190) 95.5–99.7
Fraen PPV	65.7% (69/105) 55.8–74.7	-	-	-	53.6% (37/69) 41.2–65.7	90.0% (9/10) 55.5–99.7	88.5% (23/26) 69.8–97.6
Fraen NPV	80.5% (392/487) 76.7–83.9	-	-	-	79.9% (151/189) 73.5–85.4	80.6% (54/67) 69.1–89.2	77.2% (187/231) 75.3–85.8
Lumin Sensitivity	38.4% (63/164) 0.9–46.3	-	-	-	46.7% (35/75) 35.1–58.6	36.4% (8/22) 17.2–59.3	29.9% (20/67) 19.3–42.3
Lumin Specificity	91.6% (392/428) 88.5–94.0	-	-	-	82.5% (151/183) 76.2–87.7	98.2% (54/55) 90.2–100	98.4% (187/190) 95.5–99.7
Lumin PPV	63.6% (63/99) 53.4–73.1	-	-	-	52.2% (35/67) 39.7–64.6	88.9% (8/9) 51.8–99.7	87% (20/23) 66.4–97.2
Lumin NPV	79.5% (392/493) 75.7–83.0	-	-	-	79.1% (151/191) 72.6–84.6	79.4% (54/68) 67.9–88.3	79.9% (187/234) 74.2–84.9

Readers 1–3 read ZN slides, readers 4–6 read LED FM slides (all LED systems for a single specimen were read by the same reader).

There were a total of 53 false positive results by any LED FM method (Table 4). Of these, 45/53 false positive results on any LED FM method were from a single reader (27/29 iLED, 32/36 Fraen, 32/36 Lumin). The majority of the false positive readings occurred in samples with either scanty readings or 1+ FM grading.

**Table 4:** False positive results.

	iLED	Fraen	Lumin
3+	0	0	
2+	1	1	3
1+	11	11	12
Scanty	17	22	18
No grading		2	3
**Total**	**29**	**36**	**36**
	21 Fraen(+), 23Lumin (+)	21iLED (+), 24Lumin(+)	24 Fraen(+), 23 iLED(+)

Excluding the reader with the highest false positive rates, the relative sensitivities of iLED, Fraen and Lumin were 36%, 36% and 31.5%, respectively, with high specificities with all methods (99.2%, 98.4%, 98.4%).

### Quality assurance

All technologists performing microscopy were trained at the same time by a single trainer and passed proficiency testing on the first attempt. Technologists had between 3 months and 4 years' experience performing ZN microscopy prior to training in FM and the shortest experience with microscopy was not associated with the poorest performance. None of the technologists had any previous experience performing FM prior to the study.

### Per reader performance sensitivities by CD4 strata compared to culture gold standard

In the HIV-infected TB suspects for whom CD4 T cell counts were available at the time of TB suspect evaluation (N = 152), the overall sensitivities for the three technologies was lowest in patients with CD4 T cell counts<100 cells/µl, and highest in those with CD4>250 cells/µl as shown in Table 5. At the lowest CD4 strata, the Fraen FM, which had previously been evaluated by technologists as the brightest field, had the highest sensitivities. The proportion of patients on antiretroviral therapy was highest in the highest CD4 strata (23.0% with<100 cells/µl, 29.9% with 101–250 cells/µl, and 47.1% with >250 cells/µl).

**Table 5:** Sensitivities compared to positive culture (LJ or MGIT) at different CD4 T cell strata.

FM mic oscope	Reader 4: iLED	Reader 4: Fraen	Reader 4: Lumin	Reader 5: iLED	Reader 5: Fraen	Reader 5: Lumin	Reader 6: iLED	Reader 6: Fraen	Reader 6: Lumin	Overall: iLED	Overall: Fraen	Overall: Lumin	Overall-4: iLED	Overall-4: Fraen	Overall-4: Lumin
CD4<100	23.0% (3/13) (5.0–53.8)	30.8% (4/13) (9.0–61.4)	30.8% (4/13) (9.1–61.4)	20.0% (1/5) (0.5–71.6)	20.0% (1/5) (0.5–71.6)	20.0% (1/5) (0.5–71.6)	30.0% (3/10) (6.7–65.2)	60.0% (6/10) (26.2–87.8)	30.0% (3/10) (6.7–65.2)	25.0% (7/28) (10.7–44.9)	39.2% (11/28) (21.5–59.4)	28.6% (8/28) (13.2–48.7)	26.7% (4/15) (7.8–55.1)	46.7% (7/15) (21.2–73.4)	26.7% (4/15) (7.8–55.1)
CD4 101–250	33.3% (4/12) (10–65)	41.7% (5/12) (15.2–72.3)	33.3% (4/12) (10–65)	-	-	-	24.0% (6/25) (9.4–45.1)	28.0% (7/25) (12.1–49.4)	20.0% (5/25) (6.8–40.7)	24.4% (10/41) (12.4–40.3)	29.3% (12/41) (16.1–45.5)	21.9% (9/41) (10.6–37.6)	20.7% (6/29) (8.0–39.7)	24.1% (7/29) (10.3–43.5)	17.2 % (5/29) (5.8–35.8)
CD4>250	53.1% (25/47) (38.0–67.9)	55.3% (26/47) (40.1–69.8)	53.1% (25/47) (38.1–67.9)	66.7% (8/12) (34.9–90.0)	66.7% (8/12) (34.9–90.0)	58.3% (7/12) (27.7–84.8)	41.7% (10/24) (22.1–63.3)	33.3% (8/24) (15.6–55.3)	33.3% (8/24) (15.6–55.3)	51.8% (43/83) (40.6–62.9)	50.6% (42/83) (39.3–61.8)	48.1% (40/83) (37.1–59.4)	50.0% (18/36) (32.9–67.0)	44.4% (16/36) (27.9–61.9)	41.7% (15/36) (25.5–59.2)
Overall	44.4% (32/72) (32.7–56.6)	48.6% (35/72) (36.7–60.7)	45.8% (33/72) (34.0–58.0)	42.9% (9/21) (21.8–66.0)	42.9% (9/21) (21.8–66.0)	38.1 % (8/21) (18.1–61.6)	32.2% (19/59) (20.6–45.6)	35.6% (21/59) (23.6–49.1)	27.1 % (16/59) (16.3–40.2)	39.5% (60/152) (31.6–47.7)	42.8% (65/152) (34.8–51.0)	37.5% (57/152) (29.8–45.7)	35.0% (28/80) (24.7–46.4)	37.5% (30/80) (26.9–49.0)	30.0% (24/80) (20.2–41.2)

Empty cells under reader 5 show that there were 13 patients and all tested negative for all 3 tests (i.e. iLed, Lumin, and Fraen). Overall-4 represents the overall sensitivities of readers 5 & 6 excluding reader 4 who had the highest false positivity rate. Table includes only those patients with a CD4 count record.

## Discussion

In an urban HIV clinic setting, laboratory technicians could be trained to use LED FM microscopy with universally higher sensitivities by all LED FM methods compared to ZN, and high specificity. However, in our study done in a relatively resourced setting within the Infectious Diseases Institute clinic laboratory, similarly trained and proficiency tested technicians displayed considerable variability. There was a marked difference in the specificity of LED FM between the three readers, with one reader generating the vast majority of false positive readings. The errors made by this reader were independent of the LED system being used and the false positive rate was similarly high for all methods. In contrast, the other two readers maintained high specificity when using all LED FM methods (>98%). The high false positivity rate for the single reader occurred despite an initial intensive training course and a refresher training, as well as the requirement to pass a proficiency test panel prior to starting the study. These findings highlight the importance of instituting careful quality assurance measures when a new technology is introduced. Slides should be re-read by experienced technicians with feedback given on discrepant results in real time. Similarly, in Zambia, a high-incidence, resource-limited setting, a study of 16 technicians' proficiency after training which also compared all 3 microscopes showed significant differences among technicians that led to low inter-rater reliability and high misclassification rates that overwhelmed any differences in sensitivity among the LED platforms [14].

The low sensitivity of ZN (31.1%) in this population of HIV-positive TB suspects may be due to increased uptake of the intensified case finding criteria during this time period which increased the number of TB suspects tested. This may have increased the number of patients screened at an earlier stage before reaching very advanced disease, hence the high proportion of smears that are low or very low positive on ZN [15]. In contrast, much higher sensitivity was seen in our first study in which sputum was collected from TB suspects whose sputum was sent for routine investigations at Mulago Hospital laboratory where patients present late in the course of illness and are more likely to be smear-positive. Sensitivity of ZN in this population was 60.0% while sensitivity of LED-based methods varied between 63.6 and 69.1% when compared to culture. In addition, the smear gradings for the positive specimens were substantially higher than in the present study [9]. Others have also noted that FM sensitivity is comparable to ZN in HIV-infected TB suspects, but the overall decreased cost due to time savings for the laboratory technicians both in times spent staining and reading slides supports the WHO policy statement to roll-out FM microscopy [16].

Limited case finding benefit in HIV-infected patients due to low sensitivity highlights the need for improved diagnostics beyond FM, particularly in patients with low CD4 T cell counts in whom the sensitivity of LED FM was particularly low with all methods [17], [18]. Although the durable, inexpensive platform makes LED technology implementable even in areas with unreliable electricity, in HIV-infected patients, a significant number of culture-positive patients will not be detected. This is especially true if intensive case finding efforts are expanded and thereby increase the number of TB suspects to be screened by smear microscopy. We are currently evaluating whether the introduction of intensive case finding and FM screening increases the number of TB cases started on treatment, or whether implementation of GeneXpert at these sites could be justified in terms of additional case finding benefit [19].

FM will most benefit urban clinics in high-burden TB countries by reducing the time required to screen slides, particularly the low positive slides which take longer to screen using LM. However, if high specificity is not maintained, the positive predictive value of the test will be negatively impacted. This highlights the critical need for intensive quality control during the initial period of LED FM implementation and once again emphasizes the critical human factor in the quality of microscopy. Reader-to-reader variability remains a notorious problem with microscopy. When comparing various TB diagnostics, tests that rely on subjective interpretation will have more operator-to-operator variability than self-contained automated assays such as GeneXpert. Furthermore, the low sensitivity of FM for HIV-positive individuals, particularly those with low CD4 T cell counts, will limit the number of additional patients found in countries with high rates of co-infection.
